# Discovery and Validation of Barrett's Esophagus MicroRNA Transcriptome by Next Generation Sequencing

**DOI:** 10.1371/journal.pone.0054240

**Published:** 2013-01-23

**Authors:** Ajay Bansal, In-Hee Lee, Xiaoman Hong, Sharad C. Mathur, Ossama Tawfik, Amit Rastogi, Navtej Buttar, Mahesh Visvanathan, Prateek Sharma, Lane K. Christenson

**Affiliations:** 1 Division of Gastroenterology and Hepatology, Veterans Affairs Medical Center, Kansas City, Kansas, United States of America; 2 University of Kansas Medical Center, Kansas City, Kansas, United States of America; 3 Kansas Cancer Institute, University of Kansas Medical Center, Kansas City, Kansas, United States of America; 4 Bioinformatics Core Facility, University of Kansas, Lawrence, Kansas, United States of America; 5 Department of Molecular and Integrative Physiology, University of Kansas Medical Center, Kansas City, Kansas, United States of America; 6 Department of Pathology, Veterans Affairs Medical Center, Kansas City, Kansas, United States of America; 7 Department of Gastroenterology, Mayo Clinic, Rochester, Minnesota, United States of America; Sun Yat-sen University, China

## Abstract

**Objective:**

Barrett's esophagus (BE) is transition from squamous to columnar mucosa as a result of gastroesophageal reflux disease (GERD). The role of microRNA during this transition has not been systematically studied.

**Design:**

For initial screening, total RNA from 5 GERD and 6 BE patients was size fractionated. RNA <70 nucleotides was subjected to SOLiD 3 library preparation and next generation sequencing (NGS). Bioinformatics analysis was performed using R package “DEseq”. A p value<0.05 adjusted for a false discovery rate of 5% was considered significant. NGS-identified miRNA were validated using qRT-PCR in an independent group of 40 GERD and 27 BE patients. MicroRNA expression of human BE tissues was also compared with three BE cell lines.

**Results:**

NGS detected 19.6 million raw reads per sample. 53.1% of filtered reads mapped to miRBase version 18. NGS analysis followed by qRT-PCR validation found 10 differentially expressed miRNA; several are novel (-708-5p, -944, -224-5p and -3065-5p). Up- or down- regulation predicted by NGS was matched by qRT-PCR in every case. Human BE tissues and BE cell lines showed a high degree of concordance (70–80%) in miRNA expression. Prediction analysis identified targets that mapped to developmental signaling pathways such as TGFβ and Notch and inflammatory pathways such as toll-like receptor signaling and TGFβ. Cluster analysis found similarly regulated (up or down) miRNA to share common targets suggesting coordination between miRNA.

**Conclusion:**

Using highly sensitive next-generation sequencing, we have performed a comprehensive genome wide analysis of microRNA in BE and GERD patients. Differentially expressed miRNA between BE and GERD have been further validated. Expression of miRNA between BE human tissues and BE cell lines are highly correlated. These miRNA should be studied in biological models to further understand BE development.

## Introduction

Chronic gastroesophageal reflux disease (GERD) is an important risk factor for the development of Barrett's esophagus (BE). BE is the dominant pre-malignant lesion for esophageal adenocarcinoma [1]. The prevalence of GERD has increased substantially over the past decade with weekly reflux symptoms increased by ∼50% and will significantly impact the future rates of BE [2]. Esophageal adenocarcinoma has already increased by 600% since 1975 [3] and the increasing prevalence of GERD and BE are likely to worsen the rates of esophageal adenocarcinoma raising a significant public health concern. Understanding factors that lead to development of BE in 10–15% of GERD patients may allow for the development of prevention strategies against this cancer by timely detection and intervention. Molecular events underlying the initiation of Barrett's metaplasia are incompletely understood but biological interactions between developmental signaling pathways and morphogenetic factors appear to play key roles [4]. MicroRNA (miRNA) regulate 20–30% of the genome by binding to the mRNA transcripts and promoting their degradation and/or inhibition of translation [5], [6]. Since a single miRNA can impact several hundred genes [5], [6], miRNA can potentially impact multiple signaling pathways and elicit large effects on a cell's phenotype integral to BE development.

To date, studies have focused on identifying miRNA associated with BE progression [7], [8], [9], [10], [11], [12], [13], [14] but miRNA differentially expressed between GERD squamous epithelium and BE columnar epithelium have not been systematically examined. While it is unknown but it is plausible that miRNA could be logical targets to study for causal relationships in BE development. Additionally, miRNA can be targeted by inhibitors and mimetics that opens novel therapeutic possibilities for BE prevention [15]. For the final goal of identifying miRNA that are not simply associated with BE but are causal to the transformation of squamous to columnar mucosa, high-throughput miRNA profiling is an initial necessary step. To characterize the miRNA transcriptome of BE, we used state of the art next generation sequencing (NGS). NGS has several significant advantages over previous methods such as reverse-transcription (RT) PCR arrays and hybridization-based microarrays including high sensitivity towards low abundant transcripts, excellent reproducibility and possibility of discovering previously unknown miRNA [16]. Our aim was to perform one of the first comprehensive investigations into defining the miRNA transcriptome of well-characterized GERD and BE patients and set the platform for further biologic characterization of specific miRNA using cellular, animal and more recently organotypic [17] models. In the study described henceforth, we were able to profile the miRNA expression of GERD and BE patients using rigorous methodology and have identified several novel miRNA such as miR-708-5p, -3065-5p, -944 and -224-5p to be associated with BE that were predicted to regulate important developmental, inflammatory and metabolic pathways.

## Methods

### Ethics Statement

The current study was approved by the Institutional Review Board of the Veterans Affairs Medical Center, Kansas City. All subjects provided written and signed informed consent. All research was conducted in accordance with the principles outlined in the Declaration of Helsinki.

### Selection of GERD and BE patients

Patients with GERD and BE were selected from a prospective tissue and serum repository (Clinical Trials.gov # NCT00574327). The Institutional Review Board of the Veterans Affairs Medical Center, Kansas City, Missouri, approved this repository. Patients presenting to the endoscopy unit for evaluation of reflux symptoms or screening/surveillance of BE were invited to participate in the study. After signing informed consent, all patients were required to fill a validated GERD questionnaire [18]. Patients with inability to provide written informed consent, advanced chronic liver disease, severe uncontrolled coagulopathy, and prior history of esophageal or gastric surgery or BE ablation were excluded from the repository.

The patients were defined to have GERD if they answered affirmative to the presence of heartburn and/or regurgitation. After endoscopic examination, GERD patients were further sub-classified into those with erosive esophagitis (EE) and those without (Non-erosive reflux disease, NERD). BE was defined as presence of columnar lined esophagus at least 1 cm in length on endoscopy with demonstration of intestinal metaplasia in biopsies. To minimize misclassification, BE patients were biopsied only if they had no evidence of active reflux disease i.e. erosions or ulcers in the Barrett's segment. Only those BE patients that did not have dysplasia were included in the current study to minimize the impact of dysplasia grade on miRNA expression. For the initial high-throughput discovery phase with NGS, only patients with a definitive diagnosis of GERD based on the presence of EE were included. In the validation phase by qRT-PCR, patients with EE as well as NERD were allowed. Research biopsies were obtained as part of a standardized protocol for collection of specimens for the tissue repository. Per protocol, in GERD patients, 2 biopsies were obtained at 1 cm and 5 cm above the gastro-esophageal junction. In BE patients, 2 biopsies were obtained every 2 cm of the BE length. Immediately after procurement, each biopsy specimen was divided into two halves- one half was randomly selected to be fixed in 10% formalin for histopathological evaluation while the other half was placed in RNAlater preservative (Applied Biosystems, Foster City, CA) for miRNA studies.

### Histologic review, RNA extraction and quality control

4 µm thick sections were stained by hematoxylin and eosin and reviewed by a single experienced gastrointestinal pathologist according to the revised Vienna classification [19]. Specimens were examined for the presence of intestinal metaplasia characterized by the presence of histologically typical goblet cells. Total RNA was extracted using Trizol as per manufacturer's protocol (Sigma, St. Louis, MO). Total RNA was quantified using a NanoDrop-1000 spectrophotometer (NanoDrop Technologies, Wilmington, DE), and quality was assessed on an Agilent 2001 Bioanalyzer (Agilent Technologies, Santa Clara, CA) and only the highest quality samples with RNA integrity number (RIN) of >8 were used for NGS. The RIN value (mean ± SEM) for the validation cohort (n = 67) was 6.9±0.7.

### Next generation sequencing

Total RNA from GERD (n = 5) and BE (n = 6) patients was size fractionated on Flash-PAGE gels and RNA (<70 nucleotides) was then subjected to SOLiD 3 library preparation [20], (Cofactor Genomics, St Louis, MO) SOLiD 3 sequencing using ligation-based sequencing technology was completed to yield 35 nucleotide reads. Reads with a minimum of 6-nucleotide adaptor sequence with no ambiguous bases and the final trimmed length of at least 15 nucleotides were included for the final alignment analysis. Median-based normalization was done. To determine the final candidate miRNA, the following steps were undertaken.

#### Step 1: Alignment to reference genome

Read sequences from NGS data were aligned into the latest version (v18) of miRBase, a repository of up-to-date miRNA information of many species including human. Alignment was performed using the bowtie short-read aligner software (version 0.12.7) [21]. Bowtie has been shown to be efficient [22] and has been successfully used in previous studies [23]. A minimum trimmed length of at least 15 nucleotides after removal of the adaptor sequences was used as previously done [11], [22]. Reads were regarded to be mature miRNA based on two conditions a) if the entire read sequence mapped within a miRNA hairpin sequence consecutively with a maximum of one mismatch and b) overlapped minimum of 7 bases to the mature miRNA. The hairpin sequence represents the precursor miRNA sequences [24] and is a unique characteristic of miRNA [25]. Reads that overlapped with multiple mature miRNA were not counted [24]. Post alignment, the number of read sequences aligned to each miRNA (read counts) was calculated. After the initial mapping to miRBase, the unmapped reads were mapped to the non-coding RNAs reported in functional RNA database (fRNAdb version 3.4), the human reference genome version 19 downloaded from UCSC genome browser (hg19) and E. coli genome allowing up to 3 mismatches. Reads that were still unmapped were remapped to the miRBase allowing 2–3 mismatches, but these reads were not used in the analysis of differentially expressed miRNA.

#### Step 2: Normalization of Read Counts

Normalization was done using the DESeq Bioconductor package in R [26] that takes the total number of reads into consideration [27]. This was done prior to the differential expression analysis to control for the variation in the number of read sequences across samples. The normalization method consisted of the following steps:

1. Construct a pseudo-reference by taking geometric mean of all miRNA. That is, the value for i-th miRNA is calculated as geometric mean of i-th miRNA in all samples.

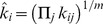
, where i = 1, …, n indexes the miRNA, j = 1, …, m indexes the sample and k_ij_ denotes the counts for i-th miRNA in j-th sample.
2. Estimate size-factor of i-th sample as the median of the ratios of the i-th sample's counts to those of the pseudo-reference's.
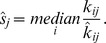

3. Divide the i-th sample's counts by its size-factor to obtain normalized counts.
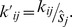

#### Step 3: Differential Expression Analysis

After normalized read counts were obtained, a state of the art statistical model for NGS differential expression analysis “R” package called DESeq [26] was used. DESeq is based on the negative binomial distribution and outputs fold change and p-values for differential expression. miRNA whose p-values (adjusted for false discovery rate of 5%) <0.05 were considered to be differentially expressed. The standard R function p.adjust was used to adjust p-values for multiple testing using the Benjamini-Hochberg method [28].

### Validation of NGS results by quantitative RT-PCR analysis in independent samples

Total RNA (50 ηg) from an additional 40 GERD patients, and 27-BE patients were reverse transcribed using hairpin RT-primers that matched our custom designed low-density qPCR array cards (Applied Biosystems). Quantitative RT-PCR was conducted using our established procedures [29], [30]. SDS software (version 2.4, Applied Biosystems) was used to identify threshold cycle (Ct) values for each PCR reaction. Expression of the small nucleolar RNAU6 was used to normalize miRNA expression measurements, and relative fold-changes of miRNA expression values between samples were calculated using the delta-delta Ct method [29]. All samples were compared to a sample from a single patient in order to calculate fold-changes. Each primer set included a minus RT control. Standard t-test was used to test differential expression of miRNA. MicroRNA with a p-value <0.05 after adjusting for the false discovery rate of 5% were labeled as differentially expressed. The standard R function p.adjust was used to adjust p-values for multiple testing using the same correction method as in NGS analysis.

### miRNA target prediction and pathway analysis

We searched the potential target genes of the miRNA and mapped the signaling pathways related to the target genes. We used multiple prediction programs including microT [31], miRanda [32], miRTarget2 [33], PicTar [34], PITA [35], RNA22 [36], and TargetScan [37]. To minimize the risk of false positives, predictions of each program were filtered by using only those scoring within the top 5%. Genes with strong prediction scores for the same miRNA from at least 2 programs were labeled as potential targets for that miRNA and used in pathway analysis. But for miRNA with no target gene shared by multiple programs, genes with prediction scores that ranked within the top 1% by any program were used as potential target genes for the miRNA. We used EGAN [38] to find KEGG pathways strongly associated with the target genes.

### miRNA expression in Barrett's cell lines

After RNA extraction, we compared the ten highest and the lowest expressed miRNA identified by NGS in human BE tissues with three different BE human cell lines, BAR-T, CP-A and CP-C. We performed this analysis to mutually validate the expression of miRNA between human BE epithelium and well established BE human cell lines and to identify appropriate cell lines for future biological experiments of miRNA modulation to understand miRNA function in BE development. BAR-T is a non-neoplastic Barrett's cell line created by hTERT immortalization of human BE cells [39]. CP-A and CP-C are immortalized cell lines created also from human Barrett's biopsies [40], [41]. CP-A expresses wild type p53 whereas CP-C hosts p53 LOH and mutations. Both CP-A and CP-C cells have p16 sequence alterations. Spearman's correlation coefficients for miRNA expression between human BE tissues and cell lines were calculated.

## Results

### Study subjects

The initial NGS cohort consisted of 11 patients, five with GERD (all with EE) and six with BE. All 11 patients were white males with mean ages of 54±4 and 61±9 years respectively. After initial NGS profiling, the miRNA were validated by qRT-PCR in independent GERD (n = 40) and BE (n = 27) patients. Mean ages were 55±13 and 61±10 years respectively. All patients were white males and were on acid suppressive therapy. Mean BE length was Prague [42] M5±3.1C3.1±1.5. Hiatus hernia was present in 63% of GERD patients versus 95% of BE patients, p<0.05. Mean body mass index BMI was similar in two groups, 32±6.6 in GERD versus 30±7.7 in BE, p = NS. Among 40 GERD patients, 20 had EE and 20 had NERD, mean ages 50±14 and 59±11 years respectively, p = NS. All EE patients had Los Angeles classification B or higher grade of esophagitis.

### Discovery project and validation

**Figure 1:**
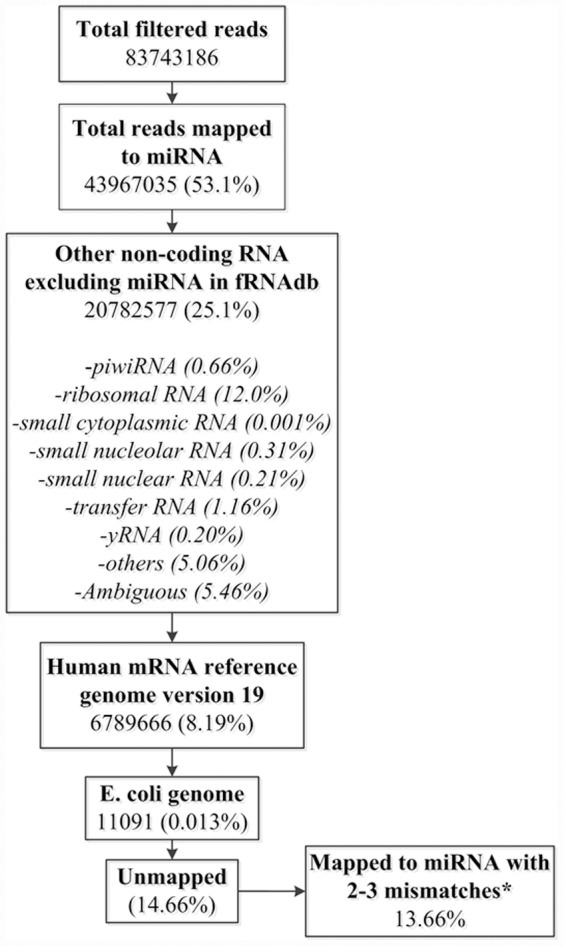
Flowchart depicting sequential mapping of the reads. As demonstrated, the unmapped reads were remapped to miRbase after relaxing the criteria to allow 2–3 mismatches leaving only ∼1% of the reads unmapped. fRNAdb, functional RNA database version 3.4; ‘ambiguous’ represents those reads that mapped to multiple different non-coding RNA in the fRNAdb; ‘others’ includes unclassified ncRNAs in fRNAdb; * these miRNA were not included in the final analysis of differential expression.

NGS completed on BE patients (n = 6) and GERD (n = 5) patients, yielded an average of 19.3 million raw reads/patient. After removing adapter sequences and filtering out reads too short to be accurately mapped (less than 15 bases), we obtained on average 7.6 million reads/patient sample and 98.5% of them were mapped to either miRBase, non-coding RNAs (fRNAdb), or human reference genome version 19 (Figure 1).

**Figure 2:**
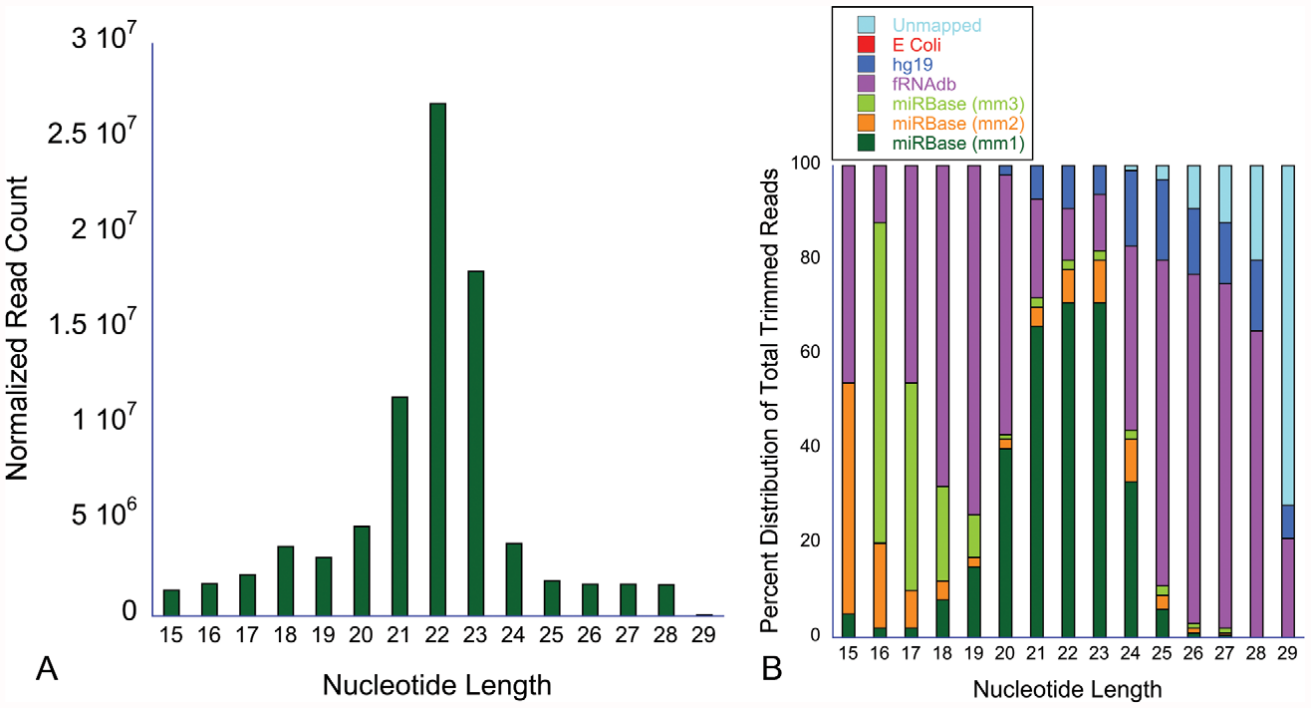
Normalized read counts and their distribution according to the nucleotide length. Fig. 1A shows that majority of trimmed reads were 21–23 nucleotides in length, the same size as miRNA. Fig. 1B shows the distribution of trimmed reads based on their mapping to miRNA, human genome, non-coding RNA (besides miRNA etc) and E coli genome. mm1, mm2 and mm3 represent alignment to miRBase with 0 or 1, 2 and 3 mismatches respectively. Note that the majority of aligned miRNA with 0 or 1 mismatch are distributed around 22 base pairs, the expected size of miRNA. hg19, human genome version 19; fRNAdb, functional RNA database.

53% of reads mapped to known miRNA in miRBase 18.0 with either 0 or 1 mismatch (Figure 1). The remaining reads were mapped to the non-coding RNA database excluding miRNA (fRNAdb version 3.4) which accounted for 25.1% of the reads of which rRNA accounted for 12%. Remaining reads were then mapped to the human genome version 19 that accounted for 8.19% of the reads. Remaining reads were also compared to the E. Coli database and a very small fraction 0.013% of reads mapped to that database. All of the unmapped reads were then remapped to the miRbase allowing for two or three mismatches (13.66% of total trimmed reads) leaving ∼1% of the reads unmapped (Figure 1). The majority of trimmed reads that mapped to miRNA were 21–23 nucleotides in length as expected for the miRNA (Figure 2A). Relative distribution of the reads into miRNA and non-miRNA databases according to read length is shown in figure 2B and again, as expected, a majority of miRNA alignment with 0 or 1 mismatch occurred between 21–23 nucleotides in length. Among 1921 known miRNA in miRBase 18, the number of miRNA detected in our samples (non-zero read counts) ranged from 736∼1122/patient (919 miRNA/patient on average). The complete list of miRNA with normalized read counts is described in Table S1. Raw NGS expression data will be made available to the investigators upon request.

### Identification of differentially expressed miRNA between GERD and BE patients

When we compared the GERD group with the BE group, 18 miRNA were differentially expressed based on DEseq FDR adjusted p value of <0.05 (Table 1). Of these 18 miRNA, four miRNA (-miRs-4253, -4776-3p, -548n and -675-3p) had reads <25 in each group and were excluded from the RT-PCR validation step. Of the 14 miRNA included in the qRT-PCR validation and evaluated in the larger cohorts of patient, all but one (miR-551b-3p) were detectable by qRT-PCR. Of the 13 detected miRNA by RT-PCR, ten were significantly different between the two groups (Table 1). Several of these miRNA are new and not been previously described to be associated with BE, such as miR-708-5p, -3065-5p, -944 and -224-5p. Of the 10 miRNA discovered by NGS and validated by qRT-PCR, three were up-regulated (log_2_ fold change 5.9–6.8) and seven were down-regulated (log_2_ fold change 2.9–6.2). Additionally, reassuringly, there was 100% consistency in the direction of fold change between NGS and RT-PCR datasets for all validated miRNA. In other words, if a miRNA was found as up-regulated by NGS data, it was also found to be up-regulated by RT-PCR and the same principle was true for down-regulated miRNA. Upon subgroup analysis, none of the evaluated miRNA were differentially expressed between the EE and NERD groups (Table 2). We also compared the BE group with the EE and the NERD subgroups (Table 2). Similar miRNA were found to be differentially expressed between the BE versus EE and the BE versus NERD groups. There were minor differences in the degree of fold change between the BE/EE and BE/NERD groups without any statistical significance.

**Table 1:** Differential expression of miRNA between BE and GERD.

miRNA	Discovery phase (NGS)		Validation phase (RT-PCR)	
	Fold change^1^ (BE/GERD)	p-value^2^	Fold change^3^ (BE/GERD)	p-value^2^
*hsa-miR-194-5p*	6.83	9.11E-08	6.5	2.18E-14
*hsa-miR-215*	5.85	1.78E-06	9.6	1.30E-12
*hsa-miR-205-5p*	-4.72	2.06E-05	−10.0	4.35E-11
*hsa-miR-192-5p*	5.58	3.99E-05	7.6	4.05E-16
*hsa-miR-203*	-4.73	3.99E-05	−4.4	1.97E-15
*hsa-miR-944*	-6.24	8.44E-04	−10.1	3.80E-11
*hsa-miR-224-5p*	-3.37	1.27E-03	−7.9	3.72E-07
*hsa-miR-708-5p*	-3.66	2.39E-03	−3.7	6.21E-07
*hsa-miR-338-3p*	4.28	2.83E-03	1.67	1.0
*hsa-miR-149-5p*	-2.88	2.83E-03	−4.4	2.46E-12
*hsa-miR-1260b*	-3.41	3.43E-03	−0.79	0.07
*hsa-miR-551b-3p*	4.22	3.43E-03	ND	NA
*hsa-miR-3065-5p*	-3.9	0.01	−1.8	0.001
*Has-miR-196a-5p*	3.97	0.03	0.08	1.0

NGS, next generation sequencing; BE, Barrett's Esophagus, GERD, Gastroesophageal reflux disease; negative numbers indicate downregulation in BE compared to GERD;

1 fold changes were expressed as log_2_ values;

2 p-value was adjusted for false discovery rate of 5%;

3 fold change by RT-PCR calculated by ΔΔCt method;

miRs-4253, -4776-3p, -548n and -675-3p were also significantly different but with reads <25 in each group and were not included for RT-PCR validation.

**Table 2:** Differentially expressed miRNA in BE compared to the GERD subgroups, EE and NERD.

miRNA	BE/EEFold change^1^(p-value^2^)	BE/NERDFold change^1^(p-value^2^)	EE/NERDFold change^1^(p-value^2^)
*hsa-miR-194-5p*	7.1 (1.39E-11)	5.9 (3.70E-06)	−1.2 (1.0)
*hsa-miR-215*	9.8 (1.34E-07)	9.1 (3.85E-05)	−0.6 (1.0)
*hsa-miR-205-5p*	−9.7 (1.85E-10)	−10.2 (3.95E-11)	−0.5 (1.0)
*hsa-miR-192-5p*	8.8 (4.33E-10)	6.4 (9.80E-07)	−2.5 (0.87)
*hsa-miR-203*	−4.2 (1.48E-14)	−4.6 (2.19E-15)	−0.4 (1.0)
*hsa-miR-944*	−10.1 (2.47E-11)	−10.1 (2.15E-11)	−0.05 (1.0)
*hsa-miR-224-5p*	−7.6 (1.07E-06)	−8.2 (1.86E-07)	−0.6 (1.0)
*hsa-miR-708-5p*	−3.5 (8.07E-06)	−4.0 (7.26E-07)	−0.5 (1.0)
*hsa-miR-338-3p*	1.8 (1.0)	1.6 (1.0)	0.2 (1.0)
*hsa-miR-149-5p*	−3.9 (4.33E-10)	−4.9 (1.48E-08)	−0.97 (1.0)
*hsa-miR-1260b*	−0.75 (0.4)	−0.83 (0.13)	−0.07 (1.0)
*hsa-miR-551b-3p*	ND	ND	ND
*hsa-miR-3065-5p*	−1.4 (0.03)	−2.2 (0.002)	−0.74 (1.0)
*hsa-miR-196a-5p*	0.23 (1.0)	0.07 (1.0)	−0.31 (1.0)

BE, Barrett's esophagus; GERD, Gastroesophageal reflux disease; EE refers to those GERD patients with erosive esophagitis; NERD (Non-erosive reflux disease) refers to those GERD patients without erosive disease; NGS, next generation sequencing; negative numbers indicate downregulation in BE compared to GERD;

1 Fold changes by RT-PCR calculated by ΔΔCt method;

2 p-value was adjusted for false discovery rate of 5% and reported in parentheses; miRs-4253, -4776-3p, -548n and -675-3p were also significantly different but with reads <25 in each group and were not included for RT-PCR validation; ND, not detected.

### Genes targeted by the identified miRNA

**Figure 3:**
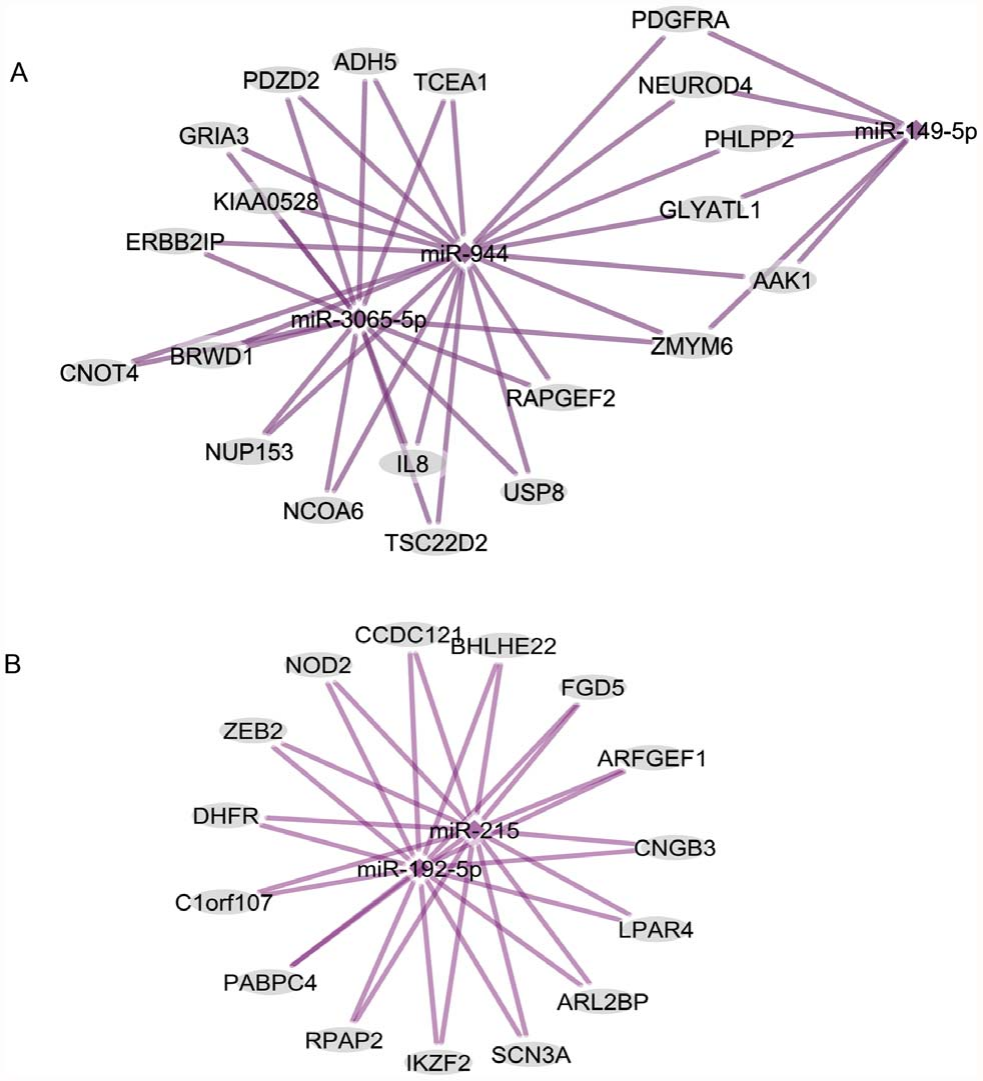
Prediction of target genes for similarly regulated miRNA. Note that miR -3065, -944 and -149-5p all were down-regulated and share multiple common targets (Panel A) and miR-192 and -215 all were up-regulated and share common targets (Panel B). These results of common targets for similarly up- or down- regulated miRNA suggest coordination between miRNA.

Only those targets that scored in the top 5% of all predictions by at least two different programs or scored in the top 1% by any one program were included. Using these criteria, targets for the differentially expressed miRNA between BE and GERD group were identified (Table S2). These targets belonged to multiple signaling pathways including TGFβ, MAPK, Notch, mTOR, WNT, hedgehog and PPAR, several of which regulate embryological development and differentiation. On further analysis, several of the miRNA shared common targets and were similarly up- or down regulated (Figure 3). For instance, miRs-3065, -149 and -944 shared common targets and were similarly downregulated (Fig. 3A). Note that two of these, miR -3065 and -944 are new and not previously described in association with BE. Similarly, miR -192 and -215 shared common targets and were both upregulated (Figure 3B). These miRNA-mRNA target analyses suggest a coordinated interplay between several miRNA in regulation of target genes that may play a role in the development of BE and their role in BE genesis need to be further validated.

### miRNA expression in Barrett's cell lines

We examined the expression of the ten most over- (miR-192-5p, 103a-5p, 145-5p, -215, -451a, -23b-3p, -21-5p, 23a-3p, 24-3p, 191-5p) and under- expressed (miR-491-3p, -574, -18a, -488-5p, -216a, -548, -520d, -20b, -218, -346) human BE miRNA in three BE cell lines, BAR-T, CP-A and CP-C. The mean number of reads by NGS for the ten most expressed miRNA in human BE specimens was 78178 (range 27,374–240,611). Of these 10 miRNA highly expressed in human BE tissues, eight were expressed in the BAR-T cell line, mean Ct 25.7 (range 18–31) and seven were expressed in CP-A and CP-C cell lines, mean Ct cycles 24.9 (range 17–31) and 24.7 (18–30) respectively. miR-215 that was highly expressed in human BE tissues was expressed only in the BAR-T cell line. The mean number of copies by NGS for the ten most under-expressed miRNA in human BE specimens was 1.04 (range 1.00–1.05). Seven of these were not detected in any of the three cell lines, all Ct greater than 40. The correlation coefficients for miRNA expression with human BE tissues were higher for BAR-T than the CP-A and CP-C cell lines, Spearman's rho −0.71, −0.59 and −0.55 respectively, all p<0.05, negative sign denotes inverse relationship between copy number and Ct cycle. Thus, there was a significant degree of concordance in miRNA expression between human BE tissues and BE human cell lines.

## Discussion

MicroRNA can regulate multiple genes and impact multiple cellular processes including cell fate and differentiation [6], [43] and likely regulate the development of BE. To the best of our knowledge, this is the first study that has comprehensively examined the GERD and BE miRNA transcriptome using NGS. This study not only confirmed previously known BE associated miRNA shown in small studies, we also discovered new miRNA potentially associated with the initiation and development of BE. To this effect, we have established a list of miRNA up- and down-regulated between well-defined GERD and BE patients from a prospective tissue repository. We did not observe significant differences in fold changes of miRNA when BE patients were compared with the GERD subgroups, EE and NERD. Our findings that majority of differentially expressed miRNA were down-regulated in BE is consistent with the proposed role of miRNA as oncosuppressors and their consequent downregulation in neoplasia [44]. Additionally, the miRNA expression of BE patients correlated well with that of BE cell lines suggesting that these cell lines may be useful to further understand the role of miRNA in BE pathogenesis. Differentially expressed miRNA discovered in this study target genes that map to pathways important in embryological development and differentiation such as TGFβ, Notch, WNT, hedgehog; inflammation such as toll-like receptor signaling, TGFβ, T cell receptor signaling, chemokine signaling pathway; metabolism and survival such as mTOR; homeostatic signaling such as MAPK and lipid homeostasis such as PPAR (Table S2). Also, similarly up-regulated and down-regulated miRNA shared common targets suggesting coordination between miRNA in regulation of BE development. These miRNA should be studied further to elucidate specific miRNA regulated molecular mechanisms that lead to the development of BE in a subset of patients with chronic GERD.

Previous studies by our group and others evaluating miRNA expression in BE have focused on identification of the miRNA associated with the progression of BE to dysplasia and adenocarcinoma [7], [8], [9], [10], [11], [12], [13], [14]. These studies have identified several miRNA that are associated with the development of BE neoplasia. However, none of the studies have focused on systemic identification of miRNA associated with the squamous to columnar switch as seen in GERD patients who harbor BE. A few studies evaluated both dysplastic and non-dysplastic patients and compared them with controls using hybridization arrays and found several differentially expressed miRNA such as miR-215, -192, and miR-205 [8], [45]. Another study that compared select miRNA between paired squamous and columnar tissues from seven BE patients found miR-215 and -192 to be upregulated and miR-203 and -205 to be downregulated in the columnar epithelium [46]. Our NGS dataset not only confirmed the significantly different expression of miR-215, -192, -203 and -205 between GERD and BE but took a more comprehensive approach to identify several novel miRNA not previously described in BE, such as miR-708, -944, -224-5p, -3065-5p among others. Some of the miRNA identified in the current study have relatively low copy numbers (footnote Table 1) and thus, were uniquely identified by NGS but likely missed by microarrays due to lower sensitivity [16]. However, at this point, the relationship between copy numbers and their biologic relevance is unclear. Since a miRNA can regulate several hundred genes, a miRNA with small copy numbers could still have a significant effect on cellular processes.

We also compared the expression of the ten most over- and under- expressed miRNA in human BE tissues with three BE cell lines, BAR-T, CP-A and CP-C. There was a high degree of concordance between the miRNA expression in human BE tissues and three distinct BE cell lines. 70–80% of the over-expressed human BE miRNA were also expressed in the three BE cell lines (all Ct <31) and 70% of the least expressed human BE miRNA were not detected in any of the BE cell line (all Ct >40). miR-215 that was highly expressed in human BE tissues was expressed only in the BAR-T cell line, perhaps suggesting BAR-T to be a good cell line for biological experiments of miRNA modulation to further understand the pathways associated with BE pathogenesis.

The role of miRNA in the origin of BE remains under-evaluated but is plausible. Direct evidence for the important role of miRNA in maintenance of columnar epithelia comes from mice with the intestine specific knockout of Dicer [47], an enzyme obligatory for miRNA processing. In these mice, the intestinal epithelium was disorganized with decrease in goblet cells and increased intestinal inflammation. A recent study over-expressed miR-145 in Het-1A and BAR-T cells and showed changes in expression of important BE related genes such as BMP4 and provide rationale for miRNA involvement in BE development [45]. Software based prediction analysis of targets for the differentially expressed miRNA in this study mapped to multiple signaling pathways related to development and inflammation such as TGFβ [48], MAPK, Notch, mTOR, WNT, hedgehog, PPAR, Toll like receptor chemokine signaling several of which have been implicated in the origin of BE [49], [50]. Interestingly, miRNA-944, a novel miRNA detected in this study regulates HOXB5 (Table S2). HOXB5 is a transcription factor of the homeobox family that has recently been experimentally validated to regulate BE development [51]. Cluster analysis found multiple similarly regulated (up or down) miRNA to share common targets suggesting a coordinated interplay between miRNA in regulation of BE development (Figure 3). The majority (7/10) of validated miRNA in the current study were down-regulated in BE compared to GERD suggesting that squamous to columnar phenotype is associated with activation of previously repressed genes. MicroRNA have also been shown to modulate cellular differentiation in other organ systems [52], [53]. There are several competing theories for the origin of BE, the two prominent ones being transdifferentiation of the squamous cells [4] versus repopulation of the distal esophagus from the embryonic precursor cells at the squamocolumnar junction [54]. In both of these models, there are regulating factors other than the cell of origin that lead to the metaplastic change of BE in a subset of GERD individuals. Supported by significant differences in miRNA profiles between the GERD and BE population, we propose that the miRNA may regulate development of BE and need to be further evaluated. Previous studies have demonstrated the feasibility of molecular diagnosis of BE by measurement of Trefoil factor 3 expression on cytology specimens [55]. MicroRNA expression appears to be highly discriminative between GERD and BE patients and can similarly be useful for the molecular diagnosis of BE. Whether these miRNA can be also used as molecular markers of cancer progression remains to be seen.

The three commonly used methods for high-throughput miRNA analysis are RT-PCR arrays, hybridization-based microarray and NGS. RT-PCR arrays can only detect known miRNA. Hybridization based technologies are limited by issues related to probe design and array background [16]. NGS does not require prior knowledge of small RNA transcripts [16], allows discovery of other non-miRNA small RNA molecules such as Piwi-interacting RNAs [5]. NGS has high sensitivity towards low abundance transcripts and excellent reproducibility. A limitation of NGS [56] is that miRNA copy numbers depend on the method used for RNA library preparation [57]. However, NGS is a highly robust method for comparing relative abundance of miRNA copies across samples since any biases introduced by the preparation method are highly systematic [57]. An important determinant of the usefulness of a high-throughput methodology is its validation by standardized techniques. The validation rate for our NGS data by qRT-PCR was ∼70%, significantly higher than a rate of 30–40% reported for miRNA hybridization microarrays [58].

Our study does have limitations but we believe that they do not alter our interpretation. The sample sizes for the discovery phase were relatively small. Sample size calculations for NGS are not well defined and are dictated by cost constraints as practiced in other NGS studies [59], [60]. We would like to emphasize that close to three-fourths of the differentially expressed miRNA discovered by NGS were validated by qRT-PCR (adjusted for multiple testing) with direction of fold change matched in every case and attests to the robustness of our procedures for NGS analysis. The average alignment rates of the NGS datasets in this study were somewhat lower. However, this has also been noted in other studies on cervical cancer where both patient specimens and cell lines were sequenced and could be explained on the basis of some cellular damage during the acquisition of clinical specimens [59]. However, the alignment rates were not significantly different between the GERD and BE samples and would not affect the differentially detected miRNA. Barrett's biopsies may contain more stroma than squamous biopsies but still such biopsies are predominantly (∼90%) composed of epithelial cells as suggested by previous flow cytometry studies [61]. pH monitoring was not performed to confirm GERD. However, a validated GERD questionnaire was used at the time of recruitment. Moreover, pH monitoring is not practical as part of patient enrollment into a tissue repository and could discourage subject participation with potential for recruitment bias.

In summary, the current study has discovered and validated the miRNA transcriptome of GERD and BE patients by next generation sequencing. The results validated previously described miRNA as well as discovered novel miRNA in BE and provide a comprehensive list of miRNA to be the subject of future molecular research into the pathogenesis of BE using animal and cellular models. The target genes and the pathways being regulated by the identified miRNA need to be further deciphered.

## Supporting Information

Table S1 The table lists complete list of miRNA identified by NGS with normalized read counts in BE and GERD groups.
(XLSX)

Table S2 The table lists the potential target genes for the differentially expressed miRNA. The miRNAs with * in front of their names had no strong target gene shared by multiple programs and predictions scored in top 1% by any program is shown in the table. The column ‘Associated Pathways’ lists pathways significantly enriched among potential target genes of a miRNA. The column ‘# of supporting programs’ denotes the number of programs that predicted the genes as potential target of the miRNA. The target genes reported in literatures registered in either miRecords or TarBase are marked as ‘Literature’ in this column.
(DOC)

## References

[1] Eloubeidi MA, Mason AC, Desmond RA, El-Serag HB (2003) Temporal trends (1973–1997) in survival of patients with esophageal adenocarcinoma in the United States: a glimmer of hope? Am J Gastroenterol 98: 1627–1633. 12873590 10.1111/j.1572-0241.2003.07454.x

[2] Ness-Jensen E, Lindam A, Lagergren J, Hveem K (2011) Changes in prevalence, incidence and spontaneous loss of gastro-oesophageal reflux symptoms: a prospective population-based cohort study, the HUNT study. Gut 10.1136/gutjnl-2011-300715 22190483

[3] Pohl H, Welch HG (2005) The role of overdiagnosis and reclassification in the marked increase of esophageal adenocarcinoma incidence. J Natl Cancer Inst 97: 142–146. 15657344 10.1093/jnci/dji024

[4] Souza RF, Krishnan K, Spechler SJ (2008) Acid, bile, and CDX: the ABCs of making Barrett's metaplasia. Am J Physiol Gastrointest Liver Physiol 295: G211–218. 18556417 10.1152/ajpgi.90250.2008

[5] Ghildiyal M, Zamore PD (2009) Small silencing RNAs: an expanding universe. Nat Rev Genet 10: 94–108. 19148191 10.1038/nrg2504 PMC2724769

[6] Inui M, Martello G, Piccolo S (2010) MicroRNA control of signal transduction. Nat Rev Mol Cell Biol 11: 252–263. 20216554 10.1038/nrm2868

[7] Bansal A, Lee IH, Hong X, Anand V, Mathur SC, et al (2011) Feasibility of MicroRNAs as Biomarkers for Barrett's Esophagus Progression: A Pilot Cross-Sectional, Phase 2 Biomarker Study. Am J Gastroenterol 10.1038/ajg.2011.37 21407181

[8] Fassan M, Volinia S, Palatini J, Pizzi M, Baffa R, et al (2010) MicroRNA expression profiling in human Barrett's carcinogenesis. Int J Cancer 10.1002/ijc.25823 PMC4303574 21128279

[9] Feber A, Xi L, Luketich JD, Pennathur A, Landreneau RJ, et al (2008) MicroRNA expression profiles of esophageal cancer. J Thorac Cardiovasc Surg 135: 255–260 discussion 260. 18242245 10.1016/j.jtcvs.2007.08.055 PMC2265073

[10] Kan T, Sato F, Ito T, Matsumura N, David S, et al (2009) The miR-106b-25 polycistron, activated by genomic amplification, functions as an oncogene by suppressing p21 and Bim. Gastroenterology 136: 1689–1700. 19422085 10.1053/j.gastro.2009.02.002 PMC2887605

[11] Leidner RS, Ravi L, Leahy P, Chen Y, Bednarchik B, et al (2012) The microRNAs, MiR-31 and MiR-375, as candidate markers in Barrett's esophageal carcinogenesis. Genes Chromosomes Cancer 51: 473–479. 22302717 10.1002/gcc.21934 PMC3547654

[12] Maru DM, Singh RR, Hannah C, Albarracin CT, Li YX, et al (2009) MicroRNA-196a is a potential marker of progression during Barrett's metaplasia-dysplasia-invasive adenocarcinoma sequence in esophagus. Am J Pathol 174: 1940–1948. 19342367 10.2353/ajpath.2009.080718 PMC2671281

[13] Mathe EA, Nguyen GH, Bowman ED, Zhao Y, Budhu A, et al (2009) MicroRNA expression in squamous cell carcinoma and adenocarcinoma of the esophagus: associations with survival. Clin Cancer Res 15: 6192–6200. 19789312 10.1158/1078-0432.CCR-09-1467 PMC2933109

[14] Yang H, Gu J, Wang KK, Zhang W, Xing J, et al (2009) MicroRNA expression signatures in Barrett's esophagus and esophageal adenocarcinoma. Clin Cancer Res 15: 5744–5752. 19737949 10.1158/1078-0432.CCR-09-0385 PMC2745487

[15] Lanford RE, Hildebrandt-Eriksen ES, Petri A, Persson R, Lindow M, et al (2010) Therapeutic silencing of microRNA-122 in primates with chronic hepatitis C virus infection. Science 327: 198–201. 19965718 10.1126/science.1178178 PMC3436126

[16] Ozsolak F, Milos PM (2011) RNA sequencing: advances, challenges and opportunities. Nat Rev Genet 12: 87–98. 21191423 10.1038/nrg2934 PMC3031867

[17] Kosoff RE, Gardiner KL, Merlo LM, Pavlov K, Rustgi AK, et al (2012) Development and characterization of an organotypic model of Barrett's esophagus. Journal of cellular physiology 227: 2654–2659. 21882191 10.1002/jcp.23007 PMC3352665

[18] Locke GR 3rd, Talley NJ, Fett SL, Zinsmeister AR, Melton LJ 3rd (1997) Prevalence and clinical spectrum of gastroesophageal reflux: a population-based study in Olmsted County, Minnesota. Gastroenterology 112: 1448–1456. 9136821 10.1016/s0016-5085(97)70025-8

[19] Montgomery E, Bronner MP, Goldblum JR, Greenson JK, Haber MM, et al (2001) Reproducibility of the diagnosis of dysplasia in Barrett esophagus: a reaffirmation. Hum Pathol 32: 368–378. 11331953 10.1053/hupa.2001.23510

[20] Metzker ML (2010) Sequencing technologies - the next generation. Nat Rev Genet 11: 31–46. 19997069 10.1038/nrg2626

[21] Langmead B, Trapnell C, Pop M, Salzberg SL (2009) Ultrafast and memory-efficient alignment of short DNA sequences to the human genome. Genome Biol 10: R25. 19261174 10.1186/gb-2009-10-3-r25 PMC2690996

[22] Buermans HP, Ariyurek Y, van Ommen G, den Dunnen JT, Hoen PA (2010) New methods for next generation sequencing based microRNA expression profiling. BMC genomics 11: 716. 21171994 10.1186/1471-2164-11-716 PMC3022920

[23] Joyce CE, Zhou X, Xia J, Ryan C, Thrash B, et al (2011) Deep sequencing of small RNAs from human skin reveals major alterations in the psoriasis miRNAome. Human molecular genetics 20: 4025–4040. 21807764 10.1093/hmg/ddr331 PMC3177648

[24] Hackenberg M, Sturm M, Langenberger D, Falcon-Perez JM, Aransay AM (2009) miRanalyzer: a microRNA detection and analysis tool for next-generation sequencing experiments. Nucleic Acids Res 37: W68–76. 19433510 10.1093/nar/gkp347 PMC2703919

[25] Berezikov E (2011) Evolution of microRNA diversity and regulation in animals. Nature reviews Genetics 12: 846–860. 10.1038/nrg3079 22094948

[26] Anders S, Huber W (2010) Differential expression analysis for sequence count data. Genome Biol 11: R106. 20979621 10.1186/gb-2010-11-10-r106 PMC3218662

[27] Meyer SU, Pfaffl MW, Ulbrich SE (2010) Normalization strategies for microRNA profiling experiments: a ‘normal’ way to a hidden layer of complexity? Biotechnol Lett 32: 1777–1788. 20703800 10.1007/s10529-010-0380-z

[28] Benjamini Y, Hochberg Y (1995) Controlling the false discovery rate: a practical and powerful approach to multiple testing. Journal of the Royal Statistical Society Series B 57: 289–300.

[29] Fiedler SD, Carletti MZ, Christenson LK (2010) Quantitative RT-PCR methods for mature microRNA expression analysis. Methods Mol Biol 630: 49–64. 20300990 10.1007/978-1-60761-629-0_4

[30] Carletti MZ, Fiedler SD, Christenson LK (2010) MicroRNA 21 blocks apoptosis in mouse periovulatory granulosa cells. Biol Reprod 83: 286–295. 20357270 10.1095/biolreprod.109.081448 PMC2907287

[31] Maragkakis M, Reczko M, Simossis VA, Alexiou P, Papadopoulos GL, et al (2009) DIANA-microT web server: elucidating microRNA functions through target prediction. Nucleic Acids Res 37: W273–276. 19406924 10.1093/nar/gkp292 PMC2703977

[32] Betel D, Wilson M, Gabow A, Marks DS, Sander C (2008) The microRNA.org resource: targets and expression. Nucleic Acids Res 36: D149–153. 18158296 10.1093/nar/gkm995 PMC2238905

[33] Wang X (2008) miRDB: a microRNA target prediction and functional annotation database with a wiki interface. RNA 14: 1012–1017. 18426918 10.1261/rna.965408 PMC2390791

[34] Krek A, Grun D, Poy MN, Wolf R, Rosenberg L, et al (2005) Combinatorial microRNA target predictions. Nature genetics 37: 495–500. 15806104 10.1038/ng1536

[35] Kertesz M, Iovino N, Unnerstall U, Gaul U, Segal E (2007) The role of site accessibility in microRNA target recognition. Nature genetics 39: 1278–1284. 17893677 10.1038/ng2135

[36] Miranda KC, Huynh T, Tay Y, Ang YS, Tam WL, et al (2006) A pattern-based method for the identification of MicroRNA binding sites and their corresponding heteroduplexes. Cell 126: 1203–1217. 16990141 10.1016/j.cell.2006.07.031

[37] Friedman RC, Farh KK, Burge CB, Bartel DP (2009) Most mammalian mRNAs are conserved targets of microRNAs. Genome research 19: 92–105. 18955434 10.1101/gr.082701.108 PMC2612969

[38] Paquette J, Tokuyasu T (2010) EGAN: exploratory gene association networks. Bioinformatics 26: 285–286. 19933825 10.1093/bioinformatics/btp656 PMC2804305

[39] Jaiswal KR, Morales CP, Feagins LA, Gandia KG, Zhang X, et al (2007) Characterization of telomerase-immortalized, non-neoplastic, human Barrett's cell line (BAR-T). Diseases of the esophagus : official journal of the International Society for Diseases of the Esophagus/ISDE 20: 256–264. 10.1111/j.1442-2050.2007.00683.x 17509124

[40] Barrett MT, Pritchard D, Palanca-Wessels C, Anderson J, Reid BJ, et al (2003) Molecular phenotype of spontaneously arising 4N (G2-tetraploid) intermediates of neoplastic progression in Barrett's esophagus. Cancer Res 63: 4211–4217. 12874028

[41] Palanca-Wessels MC, Klingelhutz A, Reid BJ, Norwood TH, Opheim KE, et al (2003) Extended lifespan of Barrett's esophagus epithelium transduced with the human telomerase catalytic subunit: a useful in vitro model. Carcinogenesis 24: 1183–1190. 12807723 10.1093/carcin/bgg076 PMC1559990

[42] Sharma P, Dent J, Armstrong D, Bergman JJ, Gossner L, et al (2006) The development and validation of an endoscopic grading system for Barrett's esophagus: the Prague C & M criteria. Gastroenterology 131: 1392–1399. 17101315 10.1053/j.gastro.2006.08.032

[43] Iorio MV, Croce CM (2012) microRNA involvement in human cancer. Carcinogenesis 10.1093/carcin/bgs140 PMC3514864 22491715

[44] Lu J, Getz G, Miska EA, Alvarez-Saavedra E, Lamb J, et al (2005) MicroRNA expression profiles classify human cancers. Nature 435: 834–838. 15944708 10.1038/nature03702

[45] van Baal JW, Verbeek RE, Bus P, Fassan M, Souza RF, et al (2012) microRNA-145 in Barrett's oesophagus: regulating BMP4 signalling via GATA6. Gut 10.1136/gutjnl-2011-301061 22504665

[46] Wijnhoven BP, Hussey DJ, Watson DI, Tsykin A, Smith CM, et al (2010) MicroRNA profiling of Barrett's oesophagus and oesophageal adenocarcinoma. Br J Surg 97: 853–861. 20301167 10.1002/bjs.7000

[47] McKenna LB, Schug J, Vourekas A, McKenna JB, Bramswig NC, et al (2010) MicroRNAs control intestinal epithelial differentiation, architecture, and barrier function. Gastroenterology 139: 1654–e1651, 1654-1664, 1664, e1651. 20659473 10.1053/j.gastro.2010.07.040 PMC3156097

[48] Milano F, van Baal JW, Buttar NS, Rygiel AM, de Kort F, et al (2007) Bone morphogenetic protein 4 expressed in esophagitis induces a columnar phenotype in esophageal squamous cells. Gastroenterology 132: 2412–2421. 17570215 10.1053/j.gastro.2007.03.026

[49] Krishnadath KK (2007) Novel findings in the pathogenesis of esophageal columnar metaplasia or Barrett's esophagus. Curr Opin Gastroenterol 23: 440–445. 17545783 10.1097/MOG.0b013e32814e6b4f

[50] Souza RF, Freschi G, Taddei A, Ringressi MN, Bechi P, et al (2011) Barrett's esophagus: genetic and cell changes. Annals of the New York Academy of Sciences 1232: 18–35. 21950805 10.1111/j.1749-6632.2011.06043.x

[51] di Pietro M, Lao-Sirieix P, Boyle S, Cassidy A, Castillo D, et al (2012) Evidence for a functional role of epigenetically regulated midcluster HOXB genes in the development of Barrett esophagus. Proc Natl Acad Sci U S A 10.1073/pnas.1116933109 PMC3384195 22603795

[52] Shu M, Zhou Y, Zhu W, Zhang H, Wu S, et al (2011) MiR-335 is Required for Differentiation of Malignant Glioma Cells Induced by Activation of cAMP/PKA Pathway. Molecular pharmacology 10.1124/mol.111.076166 22172575

[53] Sugatani T, Hruska KA (2009) Impaired micro-RNA pathways diminish osteoclast differentiation and function. The Journal of biological chemistry 284: 4667–4678. 19059913 10.1074/jbc.M805777200 PMC2640963

[54] Wang X, Ouyang H, Yamamoto Y, Kumar PA, Wei TS, et al (2011) Residual embryonic cells as precursors of a Barrett's-like metaplasia. Cell 145: 1023–1035. 21703447 10.1016/j.cell.2011.05.026 PMC3125107

[55] Kadri SR, Lao-Sirieix P, O'Donovan M, Debiram I, Das M, et al (2010) Acceptability and accuracy of a non-endoscopic screening test for Barrett's oesophagus in primary care: cohort study. BMJ 341: c4372. 20833740 10.1136/bmj.c4372 PMC2938899

[56] Kawaji H, Hayashizaki Y (2008) Exploration of small RNAs. PLoS Genet 4: e22. 18225959 10.1371/journal.pgen.0040022 PMC2213711

[57] Linsen SE, de Wit E, Janssens G, Heater S, Chapman L, et al (2009) Limitations and possibilities of small RNA digital gene expression profiling. Nat Methods 6: 474–476. 19564845 10.1038/nmeth0709-474

[58] Koshiol J, Wang E, Zhao Y, Marincola F, Landi MT (2010) Strengths and limitations of laboratory procedures for microRNA detection. Cancer epidemiology, biomarkers & prevention : a publication of the American Association for Cancer Research, cosponsored by the American Society of Preventive Oncology 19: 907–911. 10.1158/1055-9965.EPI-10-0071 PMC2852469 20332265

[59] Lui WO, Pourmand N, Patterson BK, Fire A (2007) Patterns of known and novel small RNAs in human cervical cancer. Cancer Res 67: 6031–6043. 17616659 10.1158/0008-5472.CAN-06-0561

[60] Persson H, Kvist A, Rego N, Staaf J, Vallon-Christersson J, et al (2011) Identification of new microRNAs in paired normal and tumor breast tissue suggests a dual role for the ERBB2/Her2 gene. Cancer Res 71: 78–86. 21199797 10.1158/0008-5472.CAN-10-1869

[61] Reid BJ, Sanchez CA, Blount PL, Levine DS (1993) Barrett's esophagus: cell cycle abnormalities in advancing stages of neoplastic progression. Gastroenterology 105: 119–129. 8514029 10.1016/0016-5085(93)90017-7

