# Supplementary material for: Discovery and Validation of Barrett's Esophagus MicroRNA Transcriptome by Next Generation Sequencing
Source: PLoS One. 2013 Jan 23;8(1):e54240. doi: 10.1371/journal.pone.0054240 (PMC3553128; doi:10.1371/journal.pone.0054240)
Supplement: Table S2 — The table lists the potential target genes for the differentially expressed miRNA. The miRNAs with * in front of their names had no strong target gene shared by multiple programs and predictions scored in top 1% by any program is shown in the table. The column ‘Associated Pathways’ lists pathways significantly enriched among potential target genes of a miRNA. The column ‘# of supporting programs’ denotes the number of programs that predicted the genes as potential target of the miRNA. The target genes reported in literatures registered in either miRecords or TarBase are marked as ‘Literature’ in this column. (DOC) [file pone.0054240.s002.doc]

**Table S2**. The list of potential target genes for the differentially expressed miRNA.

The miRNAs with * in front of their names had no strong target gene shared by multiple programs and predictions scored in top 1% by any program is shown in the table. The column ‘Associated Pathways’ lists pathways significantly enriched among potential target genes of a miRNA. The column ‘# of supporting programs’ denotes the number of programs that predicted the genes as potential target of the miRNA. The target genes reported in literatures registered in either miRecords or TarBase are marked as ‘Literature’ in this column.

| **miRNA** | **Potential Target Genes** | | **Associated Pathways (p-value)** |
| --- | --- | --- | --- |
|  | # of supporting programs | Gene Names |  |
| **miR-149-5p** | 4 | FRMD4A, SLC9A8, APOL4 | Calcium signaling pathway (0.0036), GnRH signaling pathway (0.013), Pentose phosphate pathway (0.045) |
|  | 3 | CACHD1, PRR15L, RAB43, FRMD7, GLYATL1, UPF2, N4BP3, SLC4A4, IGFBP5, GFER, CYB5B, STRA6, ADCY1 |  |
|  | 2 | RETSAT, CHCHD3, SEMA5B, DPM3, FXYD5, CSNK1G1, MRPL17, CD109, FKRP, TMEM86A, RNF138, FOXK1, C7orf41, EBP, ZNF445, UBTD2, RELT, TMEM186, NSL1, NRIP2, VASH1, POTEH, ADAMTS10, CLEC2D, DNAJC18, IBA57, GTPBP1, RNF4, PDGFRA, PRPS2, PRKCA, KCNJ5, IFNAR2, NTRK3, FCAR, RNASE11, AAK1, SEPN1, SMC1A, NDST1, SLC35B4, C17orf81, ORAI2, NEUROD4, XIRP2, CCNI, PHLPP2, ARHGEF18, MAGEB18, ZMYM6, PPIP5K1, RNF2, EGLN3, RSBN1L, STAC, ARRDC2, C22orf13 |  |
| ***miR-192** | 1 | DLL1, EBF2, KAT2B, CHMP4C, ATP6V1G2, C1orf186, MAP7, C16orf87, TCEA1, BNIP2, ZCWPW2, ZNF608, BANK1, CRY1, USO1, C14orf101, LOC728554, AMFR, C12orf11, STAG2, ARHGAP11A, PKHD1, RYK, CC2D2A, TMEM168, SNORA24, STK39, CSAD, TAPT1, POU4F2, MET, TOX, ACTR3, THBS4, CASD1, SMAD7, C4orf42, BTBD6, KIAA0947, FUT11, THOC3, AKAP6, CYP4B1, FKBP1B, MYCBP2, RALGAPA2, COX7B, ATF7IP, PTPRM, SYNCRIP, ALG8, SON, PHTF2, TFDP1, AGXT2L2, ZNF254, MAPRE2, DLEU2L, SLC25A14 | TGF-beta signaling pathway (0.00023), Notch signaling pathway (0.0020) |
| **miR-194-5p** | 4 | STAU2 |  |
|  | 3 | C9orf150, C7orf58, HBEGF, FMR1, DCUN1D4, KIAA1210 |  |
|  | 2 | BTF3L4, VN1R1, RNASE6, AGK, C2orf90, ZBBX, THAP10, SLCO1B1, TMEM136, RAB3B, ATP2C1, C14orf101, C14orf43, C7orf60, SLK, DARS, VASH2, EPC2, SLC1A3, SETD5, RUFY2, MRPL15, VPS13B, COMMD9, PER3, ATP6V1H, DOCK8, KHDRBS2, C18orf32, NVL, LUC7L3, C3orf23, CUL4B, CHD1, CXCL3, ZNF518A, FAM63B, TMBIM4, MITF, BNC1, HECTD2, USP6NL, FZD6, PPT1, BNIP2, PAIP2, NIPSNAP3B, ANGPTL1, BTBD7, ZNF615, CNTNAP2, PPFIBP1, TMED5, LRCH2, MAP2, ZKSCAN2, FBXW2, ZNF527, C11orf41, TUBB1, TAF4, PHF20, SF3B4 |  |
| **miR-203** | Literature | ABL1, TP63, SOCS3 | Metabolic pathways (0.0035), MAPK signaling pathway (0.0038), Neurotrophin signaling pathway (0.0051), mTOR signaling pathway (0.033), Insulin signaling pathway (0.039) |
|  | 5 | SLC4A4 |  |
|  | 4 | ZNF281, D4S234E, PCLO, MBNL2 |  |
|  | 3 | TMEM69, PAQR3, ADK, AQP4, KIF2A, PRPS2, ABCE1, RP2, SEC62, MPDZ, SEMA5A, VAPA, SOCS6, ONECUT2, ADAMTS6, AFF4, DNAJC27, DPPA4, SCYL2, EIF5A2, COL21A1, RELT, DCP2, SPOPL, TMTC2, GABRA1, MEX3C, ZMYM2, PPP1R12A, PDE4D, PLD1, SP4 |  |
|  | 2 | COPS7B, FAM180A, FLG2, C14orf45, PDGFD, LCOR, ADPGK, FSHR, GABRA4, ACADSB, MAP4K3, GPR18, FLRT2, CCDC19, TNRC6B, ACVR2A, ATP5G3, COL4A4, LIFR, NOVA1, ZNF148, PHLDA1, ZNF292, SOSTDC1, C7orf42, SYT13, RFX7, ALG10B, MMAA, UBN2, HS6ST3, DIAPH2, ATF2, RTKN2, PRICKLE2, HOOK1, VASH2, RAB22A, KIAA1211, MKL2, NUFIP2, TRPS1, RBM39, BEND4, IGF1R, KITLG, TADA2B, SPARC, C9orf93, C1orf96, ZC3H12B, PSD3, CRISP1, CD53, BCL7A, C2orf80, UBR1, RFX6, HNRNPUL2, TDRD6, FGG, S1PR1, EPYC, DUSP5, DGKB, COCH, CSN2, COL17A1, CREM, CCR1, WFDC13, SASS6, TTN, TSHR, PTP4A1, ZFP112, PRPF18, PDHX, CD164, INPP4B, WASF1, MAP3K13, NEMF, EEF1E1, AKAP7, ZRANB2, TMPRSS11D, CNOT8, MED14, GBE1, MSTN, HCCS, OPA1, ORC2, NCAM2, SMAD1, MAP3K1, PKHD1L1, RAP2A, PSMD5, PCSK2, PRKG1, PIK3CA, ROBO1, RNASE4, SH3BGR, SOX5, SNAI2, SYT4, MAGEB18, EFHA2, TMEM182, C4orf33, MB21D2, CCDC112, CEP120, DNHD1, WFDC5, RGS21, FAM204A, CLEC7A, FCF1, KIAA1383, KRT20, C10orf18, PCDHB7, ANKS1B, PRDM10, SMARCAD1, CXorf57, IFT57, RIF1, CASC1, PNMAL1, TMEM57, MYO5C, WDR33, ZNF695, FAM40B, TXNDC16, GBA3, G6PC2, GRHL3, UNC13C, OLFM3, SH2D1B, TEDDM1, FRG2, RNF141, PGAP1, NUBPL, C15orf44, KBTBD8, HPS3, HSDL2, PHF6, PCGF6, B3GNT5, ZMYM1, C6orf211, AADAC, SRA1, ZNF197, GPR64, NUDT21, MORF4L1, YWHAQ, WDR3, RAB10, DMGDH, OLA1, CCDC59, ZBTB11, DKK2, C10orf137, STEAP1, ANKRD34B, KIAA1429, TTC39A, KIAA1009, RRAS2, KIAA0776, SLC35A3, OPN3, KAT6B, GOLGA8A, WDFY3, CAMSAP1L1, LRRTM3, CAB39, OSBPL8, |  |
| **miR-205-5p** | Literature | MED1, ERBB3, PRKCE, ZEB2, INPPL1, ZEB1, VEGFA | mTOR signaling pathway (0.0067), Calcium signaling pathway (0.0087), PPAR signaling pathway (0.012), VEGF signaling pathway (0.014), ErbB signaling pathway (0.018), Metabolic pathways (0.049), Wnt signaling pathway (0.049) |
|  | 4 | PLCB1, CALCRL, NFAT5, CDK19, DMXL2, CCNJ, ZNF606, RAB11FIP1, CMTM4 |  |
|  | 3 | KCNJ16, TNFAIP8, CDH11, SORBS1, ADAMTS9, ETNK1, BTBD3, CDK14, NSF, LRP1, EPB41, ACSL1, CHN1 |  |
|  | 2 | VPS18, PAPPA2, RAPGEF2, UVRAG, ZBTB16, DBF4, HSPA13, TBX18, AAK1, SBF2, CASD1, LCOR, SLC35A1, PPP1R15B, KLF12, SLC30A8, C6orf201, LPCAT1, QKI, DLGAP2, MAGI1, MAGI2, PHF16, HSD17B11, KAT2B, MGRN1, SATB2, LIN9, TP53BP2, YES1, CLIP1, RPS6KA3, MDM4, KPNA1, SPANXN1, ERBB4, ESRRG, GABRA4, N6AMT1, DLG2, RBPMS2, SPINK13, CCDC80, C4orf39, TOR1AIP2, C6orf222, ANKRD22, CASC4, LRRK2, ENPP4, CADM1, ZNF638, C9orf40, TMEM144, MSL2, FAM35A, SLC25A21, RHPN2, GLYATL1, PJA2, HS3ST1, KALRN, SKAP2, PSMA4, NEU1, PAM, FOXF1, CYLC2, COX11, ZNF615, E2F3 |  |
| **miR-215** | Literature | TYMS, DHFR, DTL | NOD-like receptor signaling pathway (0.027) |
|  | 3 | CNGB3 |  |
|  | 2 | RPAP2, FGD5, ARL2BP, NOD2, CCDC121, LPAR4, ZEB2, BHLHE22, ARFGEF1, SCN3A, PABPC4, DIEXF, DICER1, IKZF2, KHDRBS3 |  |
| **miR-224-5p** | Literature | KLK10, KLK1, AP2M1, API5 |  |
|  | 4 | H3F3B |  |
|  | 3 | UBE2J1, SLMAP, SPPL3, ZDHHC20 |  |
|  | 2 | KIAA1267, NRBP2, PRSS33, FSTL5, C19orf47, CDKN1A, ITM2B, HNRNPU, PAX9, U2SURP, FAM49B, RNF38, C18orf25, UBXN4, CCPG1, LMBRD2, FRMD8, TXNDC6, CPNE8, ISM1, TANK, RNF13, FAM177A1, C20orf30, NKAIN2, ZNF423, NCOA6, HORMAD2, C19orf59, PCDH10, FAM40B, C8orf44, C9orf80, MARCH5, ZNF434, GGNBP2, INSC, AKD1, DNM1, EGR2, F2RL1, AFF3, C1orf9, PPAT, PPP2R1B, KLF10, UBE2D3, UTY, ZNF207, MAD2L1BP, PAPSS2, PJA2 |  |
| ***miR-3065-5p** | 1 | ATL2, JAG2, TLE1, TP53TG3, C1orf192, BRD4, FOXF2, RAPGEF2, EIF1B, ANK3, NMU, NRCAM, CSTF2, PNISR, U2AF2, NELL1, HOPX, SMARCA1, SPAG11B, SRSF7, SH2D6, GPRASP2, BAI3, NPY5R, LYSMD3, LTF, ELF2, RBMS3, ZZZ3, GTF3C3, ZNF804A, DDX26B, CAPRIN1, JMJD1C, CXCL6, DNAJB11, RBM46, WSB1, EEF1E1, NOVA2, E2F5, KIAA0825, LUZP4, C1orf94, GRIA4, BMP2K, PEX3, C5orf35, AKIRIN2, PLK4, SAMSN1, KEAP1, GSG2, ACAD9, C15orf40, LEO1, YY1, PIPOX, SLITRK4, OAS3, CXXC5, DHX36, CTTNBP2, ZNF212, ARHGAP21, PAN2, SMARCA2, CALCR, PISD, RAP1A, RHOBTB1, FOXC1, GUCY1B3, CLCN3, STK3, HNRNPUL2, NDFIP1, TSC22D2, UBE2I, RALGDS, GEMIN2, GOLPH3, MLLT4, HDGFRP3, AZIN1, SP100, RING1, KRTAP24-1, CNOT4, ANXA4, STAT4, RBM44, NFIL3, ZNF389, UBE2E1, TSG101, PRKD1, WRB, ARID4B, LPHN3, MAB21L1, ITGB5, ANKS1A, KCNMB2, PAX9, IL8, PSAT1, SLIT3, LOC554223, ACTR3BP2, SVEP1, LRP12, HSFY1P1, BRWD1, STIM2, SSB, SRRM1, FOXP2, SLC35A1, CDC5L, ZCCHC11, CHD4, NCOA6, KIAA0528, MET, KIAA0182, PDGFC, SLC25A31, SPP1, TMEM67, LRRC40, ESM1, MRPS30, KHDRBS3, LRP1B, FKBP6, ING3, LARP7, PSMD14, USP42, NR4A2, SLC44A5, PIK3C2G, CTH, CD96, CAT, BTBD8, RNASE6, ITGA1, RASA1, THADA, DOCK4, TM2D1, WTAP, NUP153, TCEA1, UBE2D2, PLAC8, EI24, SRSF3, RHOT1, CSAD, ZCWPW1, CCDC129, ANUBL1, GTPBP4, EIF3A, ACOT9, KDM3B, CYorf15B, ERBB2IP, TFAM, ZMYM6, SEMA4C, PPP2CB, MRFAP1, TRMT11, CD1E, GRIA3, SPG20, GTF2I, SC4MOL, GXYLT2, PTGES3, USP8, PDZD2, C18orf10, ADH5, CCDC150, ZFAND6, SEC24D | NOD-like receptor signaling pathway (0.032), MAPK signaling pathway (0.034) |
| **miR-708-5p** | 4 | SKA1 | NOD-like receptor signaling pathway (0.0011), B cell receptor signaling pathway (0.0016), Toll-like receptor signaling pathway (0.0029), T cell receptor signaling pathway (0.0032), Neurotrophin signaling pathway (0.0043), Insulin signaling pathway (0.0051), Chemokine signaling pathway (0.0095), MAPK signaling pathway (0.019), mTOR signaling pathway (0.040), Cytosolic DNA-sensing pathway (0.043) |
|  | 3 | PREPL, KIAA0355, NFE2L2, ASB5 |  |
|  | 2 | WSB2, C14orf105, TEX14, CBFB, IKZF4, PCDHA9, GIT2, ARHGAP44, GCNT3, ZNF235, CDC14A, SPARC, MAPK1, MPL, EN2, ALG9, MDGA1, RUFY3, SACM1L, FOXJ3, USP39, TMEM170A, GON4L, AGPAT3, ASPA, BAG1, NPY2R, IKBKB |  |
| **miR-944** | 4 | DCX, ASPH, EPHA7, FBN1, FLI1, HMGB1, NDUFS1, NEUROD1, PRRX1, UBL3, RAD23B, DYNLT3, TSNAX, SLMAP, SSPN, EBAG9, TSC22D2, PDLIM5, NFAT5, ZBTB1, KIAA1033, ZDHHC17, PHOCN, AHCTF1, EPC2, MRPL42, SNX10, ZNF107, CCRL1, TMX3, FAM135A, HRH4, CHD9, CPEB4, SGPP1, RAB33B, DCBLD2, PABPC5, GPR155, LPPR5, PIKFYVE, ARL13B, C2orf69, RTKN2, SEMA3D, RALGAPA1, KCNT2, THEMIS | Metabolic pathways (0.00056), Jak-STAT signaling pathway (0.0011), MAPK signaling pathway (0.0036), Insulin signaling pathway (0.010), PPAR signaling pathway (0.013), Chemokine signaling pathway (0.013), T cell receptor signaling pathway (0.015), Wnt signaling pathway (0.016), B cell receptor signaling pathway (0.018), TGF-beta signaling pathway (0.027), ErbB signaling pathway (0.029), mTOR signaling pathway (0.032), Calcium signaling pathway (0.032), Hedgehog signaling pathway (0.038), NOD-like receptor signaling pathway (0.049) |
|  | 3 | BMPR2, CA1, FOXN3, CHM, EXT1, F2RL1, GABRA4, GABRB2, GABRG1, GCNT1, GLRA2, GRIA3, HNF4G, HNRNPA2B1, HTR1F, IL8, ITGB8, MKLN1, MMP16, MNAT1, MTAP, PAK2, PDCL, PRH2, RP2, SCD, SGCB, TCEA1, TEP1, TRPS1, ZFY, PTP4A1, EVI5, CUL3, JRKL, CREG1, IL18R1, KAT2B, NFS1, CLDN12, USP8, ZMYM6, KL, ZNF264, QKI, ONECUT2, CREB5, SECISBP2L, KIAA0528, PIGK, ACTR2, EXOC5, TCFL5, ADAMTS5, AKAP11, TFEC, IKZF2, COBLL1, UHRF1BP1L, DCUN1D4, LPIN1, DICER1, AP4E1, SLC35A3, ZNF451, MED4, MYEF2, RRP15, PI15, FAM8A1, ESF1, OTUD6B, ENAH, ECHDC1, PCDHB4, PHTF2, PELI2, ARID1B, KIAA1468, ZDBF2, RFX7, MANEA, C15orf29, RMI1, PHC3, PGAP1, NAA50, C1orf21, MAGT1, LZIC, PCGF5, C12orf23, MEX3A, SLC2A13, C1QTNF3, SLC26A7, SOCS4, ZPLD1, FAM76B, ZNF714, TMEM65, LONRF2, MIER3, TMEM64, RICTOR, STEAP2, ZNF326, SREK1IP1, ATP11C, LUZP2, ZDHHC21, FAM174A, ZBTB41, ZC3H6, VGLL3, IYD, SULT6B1, MCART6, ANKRD20A5P, TMEM236, EFHC2, PRDM1, LYRM5, YAF2, PPARGC1A, C1orf173, GMFB, GATA6, DMD, NF1, NAIP, GRM7, SC5DL, C3orf15, KLHL20, APPL1, WAPAL, MGA, WDR17, IMPACT, BRWD1, SV2B, C1orf141, ERBB2IP |  |
|  | 2 | MMAA, PCDHB11, ACYP2, ADH5, BNIP3, BRAF, DSG2, FGF2, IGJ, LIFR, NCBP1, NOVA1, ATXN7, SLC12A2, SOS1, TPR, HIRA, ZNF236, PDE5A, MTMR6, AKAP5, WDR1, ROD1, STAM2, FUT9, GLIPR1, AAK1, PCNX, PEG10, LRRTM2, TMED7, REV1, ERAP1, CCDC76, TMEM106B, INO80D, ATG2B, UBE2W, VEZT, MRS2, NEUROD4, SLC5A7, SH3TC2, ZYG11B, L2HGDH, FAM105B, PCMTD1, ACVR1C, ALG10B, ZNF681, TTC14, GXYLT1, MACC1, PAIP2B, TMEM170B, UNC80, HSPA12A, C4orf34, ZNF614, KIAA2018, ZNF738, ARHGAP5, KIF3A, KLF12, NAMPT, SEMA3C, FGL2, MIPOL1, DNAH14, FSD1L, PPP1R15B, RAB2B, EYA4, DBT, COL19A1, PPP1R1C, CNOT4, NCAM2, MLL, TNPO1, IGF1R, ING2, ZNF295, SNTB2, SSTR1, PDGFRA, PRKG1, PTGFR, PTPN9, MGAM, NCOA1, ZMYM2, ACVR2B, GRPEL2, C5orf24, EFHA2, ZNF81, TMEM26, MBLAC2, GEMC1, CPEB2, FAM91A1, NADKD1, SLC7A11, NCOA6, PHLPP2, SLC44A1, SYNE1, NLGN1, CNKSR2, FCHO2, MDGA2, ZNF385D, KIAA1383, ZNF770, GEMIN8, RC3H2, RIC8B, BCL11A, STK17B, SLC4A7, KIAA0408, VPS36, CRNKL1, NIN, HECA, ZNF462, ERMN, SHROOM3, FAM40B, MIER1, TRERF1, PALM2-AKAP2, MAPK1IP1L, GAPT, TAPT1, CD47, MS4A1, APC, ANGPT2, ANGPT1, ATP6V1G2, BNIP3L, BDNF, EYA1, ETS2, ERG, GNAI1, GK, GJB2, GBP1, GBP3, GATM, GABRA2, FSHB, FSHR, DNAH6, DPYSL2, CYLC2, CISH, CHRNA5, COX11, NPHP1, NPY2R, TRIM37, MT1H, MMP10, ME1, CYP4F3, LRP2, IGFBP3, HTR2A, ICA1, HGF, HOXB5, HOXD13, HNMT, GPR34, SMS, SPAST, SLC8A3, SLC12A1, TAF4, TAF4B, RTN1, RHAG, PCSK2, PEX7, PIK3C2A, PIK3CG, PODXL, POU1F1, PRKACB, RFX4, PAFAH1B2, SKAP2, DDX18, EIF3J, VAMP4, SORBS2, PARG, BHLHE40, SPOP, RECK, FZD3, VIP, UTY, UBE2D1, ZNF124, ZNF33A, TNFAIP2, GBP7, SP5, ZNF300, SLFN11, NEXN, GLYATL1, ZNF439, ZNRF2, ANKRD20A4, ANO6, ANKDD1A, ANKRD34B, ZNF680, C14orf39, PTPN20A, OTOA, LSM6, GABARAPL2, ACTR1B, OPTN, C15orf43, NAALAD2, GCOM1, SLC19A2, ABCA9, TRDN, GLYAT, UGT2A1, SPIN1, TRAFD1, TBR1, GNB5, POLR3G, RGS14, RAD51AP1, SPICE1, JAKMIP1, ACADL, SPIN4, C7orf57, TMEM68, RFTN2, MDH1B, CCDC148, TMEM182, CCDC58, KCNG3, CNPY1, TRHDE, ARSK, C11orf54, TMPRSS11E, NOX1, NPHP3, PCLO, PTPN20B, CNNM1, HSPB8, FBXO8, BACE2, WDR43, PALLD, PDZD2, ABCA6, ABCB10, SASH1, SEPHS1, BTBD3, ZNF365, MAGEF1, HHIP, C5orf28, NSUN3, CHP2, DPY19L2, C12orf40, IL22, TRAT1, MBTD1, DPP8, CMTM6, MOSC2, SLC38A2, DPPA4, IFT57, CSGALNACT2, TMCO3, SLC35A5, PANK1, GPR88, CYP39A1, SS18L2, TUBE1, C6orf225, COG6, ARHGAP20, LRCH2, SLC4A10, SLC4A5, PDSS2, ADAMTSL3, OTUD7B, RBM22, CENPJ, TMEM126B, CEP72, ZNF83, ANKRD20A2, C5orf44, CCDC15, ULBP1, TMEM188, PAN3, ACSM2A, TSLP, GFM1, NKD1, SHANK3, AEBP2, ZNF684, TSC22D4, COLEC12, TCHHL1, TMEM47, NUDT12, MRPL45, THOC3, NSRP1, PCBD2, LRRIQ1, UTP15, KIAA1109, PGBD1, LSM12, C9orf100, LINGO1, TMEM200A, ZBBX, MAP9, NHEJ1, MCMBP, SPAG16, POF1B, CYBRD1, CXorf1, PLAA, CYTH1, AIMP1, CASP8AP2, NUP153, RALGPS1, MED17, NPEPPS, PJA2, TOMM70A, RAPGEF2, UBE3C, CEP57, ZNF536, ZFYVE16, KIAA0247 |  |
